# Toothache—Solidified Creosote

**Published:** 1888-11

**Authors:** 


Toothache.—Solidified Creosote.—Creosote as a dental application to painful cavities, is complicated with the inconvenience that the liquid is apt to give trouble by coming in contact with various parts of the mouth. This may be avoided by mixing it with collodion, in the proportion of 10 parts of collodion to 15 parts of creosote. The mixture forms a sort of jelly, which besides being more manageable than plain creosote, forms a varnish which seals the cavity and protects the dental nerve substance from contact with the air. [Pure carbolic acid may be used in place of creosote.]

